# Centering Symptom Burden in Patient-Oriented Clinical Trials in Kidney Disease

**DOI:** 10.34067/KID.0000000000000256

**Published:** 2023-09-28

**Authors:** Devika Nair

**Affiliations:** Division of Nephrology and Hypertension, Vanderbilt University Medical Center, Nashville, Tennessee; Vanderbilt Center for Health Services Research, Nashville, Tennessee; and Veterans Affairs Tennessee Valley Healthcare System, Nashville, Tennessee

**Keywords:** clinical epidemiology, depression, dialysis, ethnic minority, geriatric nephrology, health equity, diversity, and inclusion, patient self-assessment, patient-centered care

Symptom burden is the quantifiable number and severity of symptoms an individual experiences at any time.^1^ Physical and emotional symptom burden has been recognized as prevalent among those living with kidney disease for nearly two decades,^2^ yet centering symptom burden as the primary outcome of clinical trials in nephrology is still nascent. Recent trials targeting such symptoms with less frequently reported associations with mortality as pruritus^3^ represent a paradigm shift in nephrology trial outcome selection that prioritizes quality of life.

In this issue of *Kidney360*, Devaraj and colleagues conduct a of participants receiving hemodialysis who were screened for the randomized controlled Technology Assisted Stepped Collaborative Care trial.^4^ This impactful Technology Assisted Stepped Collaborative Care trial tested the effectiveness of 12 weekly sessions of virtual cognitive behavioral therapy without or without pharmacotherapy versus health education on symptom burden when delivered using a collaborative approach with therapists, nephrologists, and primary care physicians. In this *post hoc* analysis, investigators explored sociodemographic and structural differences in (*1*) pain, fatigue, and depressive symptom burden and (*2*) participant readiness to seek symptom treatment.

Results demonstrate that seventy-seven percent of participants reported more than one symptom; pain and fatigue were the most commonly reported (52% and 64%, respectively). Of 31 participants who reported already receiving treatment for a symptom, 29 reported still experiencing pain (94%). Among participants reporting two symptoms, pain and fatigue were the symptoms most commonly reported together. Adults who were (*1*) younger than 65 years and (*2*) male reported a significantly higher symptom burden and increased readiness for treatment, respectively. Among participants who reported readiness for symptom treatment and after controlling for race, income, illicit drug use, and social deprivation, an income of more than $60,000/yr associated with a lower odds of experiencing pain. Living in a neighborhood perceived as less walkable associated with a higher odds of experiencing depressive symptoms or fatigue.

Strengths of Devaraj and colleagues' analysis include the participation of individuals underrepresented in kidney disease research (17% of participants identified as Black, 25% as American Indian or Alaska Native, and 25% as Hispanic); the focus on such underinvestigated yet patient-prioritized symptoms secondary analysis as pain and fatigue; and the unique recognition that structural and environmental factors may play a role in the kidney disease symptom experience.

Some findings need further investigation and replication before robust conclusions can be drawn. The high odds ratios of experiencing greater symptom burden among participants reporting a low annual income or poor neighborhood walkability should be interpreted with caution. Odds ratios can overestimate the risk of common outcomes, applicable here given the high prevalence of symptom burden in this sample. Furthermore, the very wide confidence intervals flanking these odds ratios demonstrate that the true risk of symptom burden in the setting of low income or poor neighborhood walkability remains largely unknown.

Furthermore, although derived from validated instruments, single-item measures of pain and fatigue were used to measure symptom burden. Cutoff scores deemed clinically relevant (and which identified participants who screened in) were based on consensus among the investigators as opposed to associations with other health outcomes or patient preference. Pain was significantly lower among those whose chronologic age was dichotomized as 65 years or older versus younger than 65 years. This is in contrast with literature demonstrating that older adults report a high burden of nociceptive, neuropathic, and nociplastic pain^5^ and may be a sequelae of the cutoff scores used in this analysis. Finally, although participants who identified as Black reported significantly higher rates of pain and fatigue, as well as a higher symptom burden overall, interpretation of these findings is limited by the smaller number of Black participants and the inability to discern whether stresses associated with experiences of systemic racism may have explained the increased symptom burden in this group.

Overall, the results by Devaraj and colleagues highlight the need to better understand the mechanisms, predictors, and effect modifiers of symptom burden in kidney disease. Fatigue may relate to muscle performance or global exhaustion. Furthermore, many participants who reported receiving symptom treatment reported persistent pain. An individual's experience of pain is not only related to pain perception and catastrophizing but also transduction, conduction, transmission, and modulation in the nervous system.^5^ The impact of comorbidities, timing of the dialytic procedure, concurrent inflammation, sleep patterns, and psychological affect on symptoms remains largely unknown. The impact of uremia on symptom perception is important to elucidate if dialysis is part of a planned symptom treatment strategy, but this may not apply to those receiving conservative kidney management, a group with high symptom burden but underrepresented in clinical trials in kidney disease. Finally, symptoms can often coexist in clusters and by nature, exacerbate one another.^6^ Should clinicians and researchers treat the single-most bothersome symptom for a patient, or rather direct patients to therapies that may target multiple symptom clusters, such as exercise?^7^

Conclusions from recent Kidney Disease Improving Global Outcomes guidelines provide comprehensive, expert-based recommendations to durably reduce physical and emotional symptom burden in dialysis.^8^ These include (*1*) the need to not only treat symptoms but to also affect the intrusiveness of symptoms on patients' daily lives (symptom-related distress and/or symptom-related functional decline), (*2*) the need to establish the ideal frequency and mode of symptom collection data, and (*3*) the need to remain cognizant of limitations in time and personnel.

Still, some overall questions remain. In this analysis, of 896 participants screened for study entry, only 506 (57%) met eligibility criteria and completed symptom assessments. It remains unknown whether all patients with advanced kidney disease would benefit from symptom screening or whether more selective inclusion criteria would be needed to minimize survey burden. It is also unclear whether administering a measure of treatment readiness as was done in this analysis is absolutely necessary to help target symptom treatment and optimize chances for success in real-world settings. No consensus exists on what validated patient-reported measures or recall period should be used for symptom collection. It remains unclear whether symptom measures specific to kidney disease should be developed and validated because uremia may affect symptom experience. Whether and how cognitive impairment and physical frailty, both which occur at an age-accelerated rate in kidney disease, worsen symptom perception remains underinvestigated. Perhaps most importantly, the degree of improvement in symptom burden that is deemed acceptable by patients living with kidney disease has not yet been widely established.

Directing resources towards routine symptom data collection among patients with kidney disease does hold promise. Randomized controlled trials of symptom assessment without prescriptions for specific therapies in response has reduced symptom burden in adults with advanced solid tumors, presumably due to changes in treatment plans stemming from providers' increased awareness of participants' symptoms.^9^ These results have important applications to routine symptom collection among patients receiving dialysis because symptoms in this group have been shown to improve over time without an intervention.^10^ However, the ethical implications of incorporating routine symptom assessments as part of kidney care without having widely accessible, maximally effective therapies must be considered. Although there is increasing recognition in the nephrology community for the need for innovative interventions that may include complementary and alternative medicine, developing and durably implementing symptom treatment as part of routine kidney care will require sustained partnerships with clinicians and researchers in palliative medicine, psychiatry and behavioral sciences, and rehabilitation.

Symptoms are the primary reasons individuals seek health care. Devaraj and colleagues have provided novel, patient-centered, hypothesis generating data that should spur the continued development, testing, and implementation of symptom-focused interventions in nephrology.
